# Pharmacokinetic and Biodistribution Assessment of a Near Infrared-Labeled PSMA-Specific Small Molecule in Tumor-Bearing Mice

**DOI:** 10.1155/2014/104248

**Published:** 2014-04-07

**Authors:** Joy L. Kovar, Lael L. Cheung, Melanie A. Simpson, D. Michael Olive

**Affiliations:** ^1^Translational Research, LI-COR Biosciences, 4647 Superior Street, Lincoln, NE 68504, USA; ^2^Department of Biochemistry, University of Nebraska, Lincoln, NE 68588-0664, USA; ^3^SVS Consulting, Lincoln, NE 68516, USA

## Abstract

Prostate cancer is the most frequently diagnosed cancer in men and often requires surgery. Use of near infrared (NIR) technologies to perform image-guided surgery may improve accurate delineation of tumor margins. To facilitate preclinical testing of such outcomes, here we developed and characterized a PSMA-targeted small molecule, YC-27. IRDye 800CW was conjugated to YC-27 or an anti-PSMA antibody used for reference. Human 22Rv1, PC3M-LN4, and/or LNCaP prostate tumor cells were exposed to the labeled compounds. *In vivo* targeting and clearance properties were determined in tumor-bearing mice. Organs and tumors were excised and imaged to assess probe localization. YC-27 exhibited a dose dependent increase in signal upon binding. Binding specificity and internalization were visualized by microscopy. *In vitro* and *in vivo* blocking studies confirmed YC-27 specificity. *In vivo*, YC-27 showed good tumor delineation and tissue contrast at doses as low as 0.25 nmole. YC-27 was cleared via the kidneys but bound the proximal tubules of the renal cortex and epididymis. Since PSMA is also broadly expressed on the neovasculature of most tumors, we expect YC-27 will have clinical utility for image-guided surgery and tumor resections.

## 1. Introduction

Prostate cancer is the most frequently diagnosed cancer in men, affecting one in six, and is a leading cause of cancer mortality [1–4]. Current methods for prostate cancer detection include imaging by ultrasound and multiparametric MRI for guidance in biopsy procedures and single-photon emission computed tomography (SPECT) with ^111^In-capromab pendetide (Prostascint) for evaluation and management of the disease [5–7]. Dual modality imaging agents are being developed to take advantage of radionuclide sensitivity and the resolution of near infrared (NIR) fluorescence [8] for possible use in pre- and intraoperative applications. Clinically available NIR dyes (indocyanine green and methylene blue) are contrast agents with no tumor specificity [9, 10]. However, imaging resolution is increased using agents that couple strong tumor-targeting properties with sensitive detection and relatively rapid clearance [11]. Recent reports have described the successful intraoperative use of small molecule targeting agents in several types of cancer for evaluation of tumor margins, image-assisted resection, and laparoscopic removal of lymph nodes [12–18].

Effective targeting of tumor cells has been achieved by use of a variety of agents that are designed to recognize aspects of the tumor cell surface in a specific manner [11, 13, 19, 20]. Particularly promising results have been obtained by targeting prostate-specific membrane antigen (PSMA), a type II transmembrane glycoprotein also known as glutamate carboxypeptidase II, and folate hydrolase I, which is basally expressed by prostate epithelial cells and overexpressed in primary and metastatic prostate cancer [3, 4, 15]. Moreover, PSMA expression has been detected in a variety of other solid tumors, specifically and strongly associated with endothelial cells of the peritumoral and intratumoral neovasculature [3, 4, 21, 22]. Thus, PSMA is an attractive molecular target, both for advancing basic mechanistic studies of prostate cancer progression in preclinical models and for surgical applications [15, 21–23].

Available tools for PSMA targeting include monoclonal antibodies and a variety of small molecule or peptide ligands, each of which has advantages and limitations associated with its use [24]. Antibody-based probes are attractive because of the high level of specificity for the target and the picomolar-range affinities that can often be achieved. These properties, coupled with a longer circulating half-life than that of many small molecules, significantly lower the required dose for optimal detection [25]. Slower clearance (2-3 days) of monoclonal antibody probes may be favorable for presurgical routines, but this extended waiting period is then necessary before sufficiently high tumor-to-background signal is realized and surgery can be performed with confidence [11, 13, 20]. These probes are also potentially immunogenic. In contrast, small molecule probes can often be generated with nanomolar affinity ranges and rapid clearance rates that not only facilitate tissue penetration but also minimize potential toxicity resulting from exposure times [26–28]. Several groups have developed PSMA-specific small molecule urea-based compounds that have been successfully applied to optical imaging of tumor tissue [27–31].

In this report, we evaluate near infrared (NIR) fluorescence-based imaging of prostate tumor cells that were targeted by a small molecule PSMA targeting agent, YC-27. Comparisons to a commercially available anti-PSMA antibody were made to illustrate biodistribution and clearance issues. Both molecules were labeled with IRDye 800CW and characterized for specific binding to PSMA-positive cells in culture, followed by an evaluation of clearance and tumor-targeting efficacy in mice. Our results support the specificity of PSMA targeting for tumor detection, provide optimized conditions for its use in mice, and suggest benefits to the use of YC-27 as a targeting agent based on its pharmacokinetic properties.

## 2. Materials and Methods

### 2.1. Cell Culture, Materials, and Reagents

LNCaP and 22Rv1 human prostate carcinoma cells purchased from American Type Culture Collection (Rockville, MD) were maintained in RPMI 1640 medium containing 10% fetal bovine serum. PC3 M-LN4 cells, derived from PC3 human prostate adenocarcinoma cells, were kindly provided by Dr. Isaiah J. Fidler (MD Anderson Cancer Center, Houston, TX) and maintained in minimal essential medium containing 10% fetal bovine serum, sodium pyruvate, and nonessential amino acids. Human PSMA/FOLH1/NAALADase I antibody was purchased from R & D Systems (Minneapolis, MN). DAPI and TO-PRO-3 were purchased from Life Technologies Corporation (Carlsbad, CA). Rabbit polyclonal *β*-tubulin antibody was purchased from Santa Cruz Biotechnology (Santa Cruz, CA). Fluoromount Reagent and poly-D-lysine hydrobromide were obtained from Sigma-Aldrich Chemicals (St. Louis, MO) and Zeba Spin Desalting Column was from Thermo Fisher Scientific (Waltham, MA). Poly-D-lysine coated 96-well plates were purchased from BD Biosciences (Bedford, MA). Amino acid synthons, synthetic resin supports, and peptide coupling reagents (NovaBioChem) were purchased from EMD Millipore (Billerica, MA). IRDye 800CW NHS ester was provided by LI-COR Biosciences (Lincoln, NE). All other reagents for chemical synthesis, purification, and analysis were purchased from Sigma-Aldrich (Milwaukee, WI), VWR (Radnor, PA), Glen Research (Sterling, VA), and Thermo Fisher Scientific (Waltham, MA). High-performance liquid chromatography (HPLC) analysis and purification were performed on an Agilent 1100 Series HPLC with appropriate reverse-phase columns, Ultraviolet and Visible (UV/Vis) spectral analysis was performed on an Agilent 8453 Series Spectrophotometer, and low-resolution mass spectrometry (LRMS) was performed using electrospray (ES) techniques on an Agilent 1100 Series LC/MSD Trap (Agilent Technologies, Santa Clara, CA). The Odyssey Classic Infrared Imaging System, Aerius Automated Infrared Imaging System, and Pearl Impulse Small Animal Imager were provided by LI-COR Biosciences (Lincoln, NE).

### 2.2. Synthesis and Labeling of YC-27 and Anti-PSMA Antibody

The bioactive moiety of YC-27 was synthesized as previously reported [28] and labeled with IRDye 800CW NHS ester (YC-27 800CW) [28]. The analytical data for YC-27 from* de novo* synthesis were consistent with earlier reported results [28, 29] and the dye-to-protein ratio was 1 : 1. IRDye 800CW anti-PSMA antibody (PSMA 800CW) was prepared by the addition of IRDye 800CW NHS ester at 3 : 1 dye-to-antibody molar ratio. The solution was incubated for 3 h at room temperature followed by spin column purification to remove any remaining unconjugated dye. The final product dye-to-protein ratio was 1.9.

### 2.3. Microscopy

Three human prostate cancer cell lines with different levels of PSMA expression were used to evaluate localization and internalization of probes: PC3 M-LN4 (negative), 22Rv1 (low), and LNCaP (high) [32, 33]. PC3 M-LN4 and 22Rv1 cells were incubated at 37°C for 1 h with 300 nM YC-27 800CW or 1 *μ*g PSMA 800CW. Cells were rinsed and fixed with 4% formaldehyde for 20 min and nuclei stained with DAPI. Images were obtained by fluorescence microscopy using an Olympus IX81 Inverted Microscope equipped with a halogen bulb and NIR filters (EX:HQ760/40x, 790DCXR, EM:HQ830/50 m; Chroma Technology Corp., Rockingham, VT).

### 2.4. *In Vitro* Examination of YC-27 800CW in Cell Culture

Human A431, MCF7, U87 GM, PC3 M-LN4, and 22Rv1 cells were rinsed with PBS and lysed with Laemmli Sample buffer. After electrophoresis, proteins were transferred to a nitrocellulose membrane, blocked for 1 h with Odyssey Blocking Buffer, and incubated with primary antibodies at 1 : 2000 dilution (PSMA 800CW and rabbit polyclonal *β*-tubulin) for 1 h at room temperature with shaking. Proteins were detected with IRDye 680RD-conjugated goat anti-rabbit IgG secondary antibody (LICOR Biosciences) visualized on an Odyssey Infrared Imaging System (LI-COR Biosciences).

Binding and specificity of YC-27 800CW and PSMA 800CW were further evaluated by fluorescent cell-based assays. 22Rv1, PC3 M-LN4, and LNCaP cells were grown to approximately 80% confluency in a 96-well microtiter plate. Poly-D-lysine coated plates were required for LNCaP assays. Growth media were replaced with media containing increasing concentrations of PSMA 800CW only (0.01 to 5 *μ*g/mL), YC-27 800CW (0.1 nM to 1 *μ*M), unlabeled YC-27 (0.026 nM to 50 *μ*M, 1 : 5 dilution series) plus 28 nM YC-27 800CW, and YC-27 800CW (28 nM) plus 2-PMPA, a competitive inhibitor of the NAALADase activity of PSMA (0.5 nM to 50 *μ*M, 1 : 10 dilution series). All treatments were done in triplicate and incubated at 37°C in 5% CO_2_ for approximately 15 min. Wells were rinsed in 1x PBS and stopped by fixing with 4% formaldehyde solution for 20 min followed by four washes in 1x PBS + 0.02% Triton X-100 to remove unbound dye and permeabilize the cells. The plates were blocked in Odyssey Blocking Buffer (LI-COR Biosciences, Lincoln, NE) for 1 hour and incubated for an additional hour with TO-PRO-3 DNA stain (diluted 1 : 5000) for normalization of cell number. Washing steps were repeated with Odyssey Buffer + 0.02% Tween-20 and the plate was scanned with an Odyssey SA Automated Infrared Imaging System. Quantification was achieved by ratiometric analysis of the fluorescent intensities obtained from 700 nm (representing cell number) and 800 nm (representing labeled probe) channels. Binding (apparent *K*_*d*_) and competition (IC_50_) parameters were calculated by nonlinear curve fit analysis using GraphPad Prism 5 (GraphPad Software, San Diego, CA).

### 2.5. *In Vivo* Animal Imaging

Male SCID Hairless Outbred mice (SHO, Crl:SHO-*Prkdc* ^*SCID*^ *Hr* ^*hr*^) were purchased from Charles River (Willington, MA) and maintained on a purified irradiated maintenance diet (AIN-93 M) from Harlan Teklad (Madison, WI). All experimental procedures for animal use were previously reviewed and approved by the Institutional Animal Care and Use Committee at the University of Nebraska-Lincoln and conducted in accordance with the Guide for the Care and Use of Laboratory Animals published by the US National Institutes of Health.

Six-week-old mice were implanted subcutaneously with 22Rv1 (PSMA positive, right flank) and PC3 M-LN4 (PSMA negative, left flank) cells (1 × 10^6^ cells/100 *μ*L saline) and maintained until tumor size reached approximately 3 mm in diameter. At this time, tumor-bearing animals received either 1x PBS (100 *μ*L), IRDye 800CW carboxylate (1 nmol), IgG 800CW (IgG 800CW, 75 *μ*g), YC-27 800CW (1 nmol), or PSMA 800CW (75 *μ*g) injected via the tail vein. Three doses, 0.25, 0.5, and 1.0 nmol, were evaluated in mice with 22Rv1 tumors (*n* = 3 per dose). Specificity was further confirmed by inhibition with 2-PMPA. For this experiment, preinjection of 2-PMPA (2 *μ*g, intravenous administration) was followed by YC-27 800CW dose (0.5 nmole). Images were captured 24 h after injection of all compounds using the Pearl Impulse Small Animal imaging system.

### 2.6. Organ and Tissue Analysis

After final imaging, animals were sacrificed and tumors and organs were removed for imaging to confirm signal content and assess agent targeting. Tumors and organs were snap-frozen in OCT compound for cryosectioning. Sections (8 *μ*m thickness) were scanned using the Odyssey CLx imaging system to measure 800 nm fluorescence signal. The 800 nm fluorescence signal per pixel was used to compare targeting agent specificity and retention in tissues.

## 3. Results

### 3.1. Western Blot Analysis and Microscopic Examination

**Figure 1:**
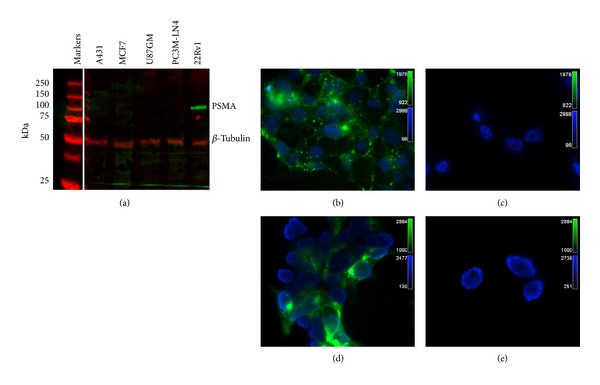
Western blot and probe localization. Human A431, MCF7, U87 GM, PC3 M-LN4, and 22Rv1 cell lysates were prepared for western blot analysis (a) to confirm PSMA reactivity of anti-PSMA antibody candidate. PSMA 800CW distinguished between 22Rv1 (PSMA positive) and PC3 M-LN4 (PSMA negative) prostate tumor cell lysates, with no signal detected for cell lysates of nonprostate origin (A431, MCF-7, and U-87 MG). Microscopy images captured cell binding for PSMA 800CW ((b) and (c)) and YC-27 800CW ((d) and (e)) to 22Rv1 (PSMA positive, (b) and (d)) and PC3 M-LN4 (PSMA negative, (c) and (d)). Images were obtained by fluorescence microscopy using an Olympus IX81 Inverted Microscope.

We first confirmed the selectivity of the PSMA-specific antibody chosen for our study reference using western analysis (Figure 1(a)). PSMA protein (85 kDa) was specifically detected in the PSMA-expressing cell line, 22Rv1. No significant nonspecific binding was noted for the nonexpressing cell lysates. Prostate tumor cell lines that were positive or negative for PSMA expression (22Rv1 and PC3 M-LN4, resp.) were incubated with PSMA 800CW (1 *μ*g, Figures 1(b) and 1(c)) or YC-27 800CW (300 ng, Figures 1(d) and 1(e)) and counterstained with DAPI to visualize nuclei. Both probes bound the PSMA-expressing cell lines and showed negligible binding to the nonexpressing cell line.

### 3.2. Cellular Specificity of YC-27 800CW

**Figure 2:**
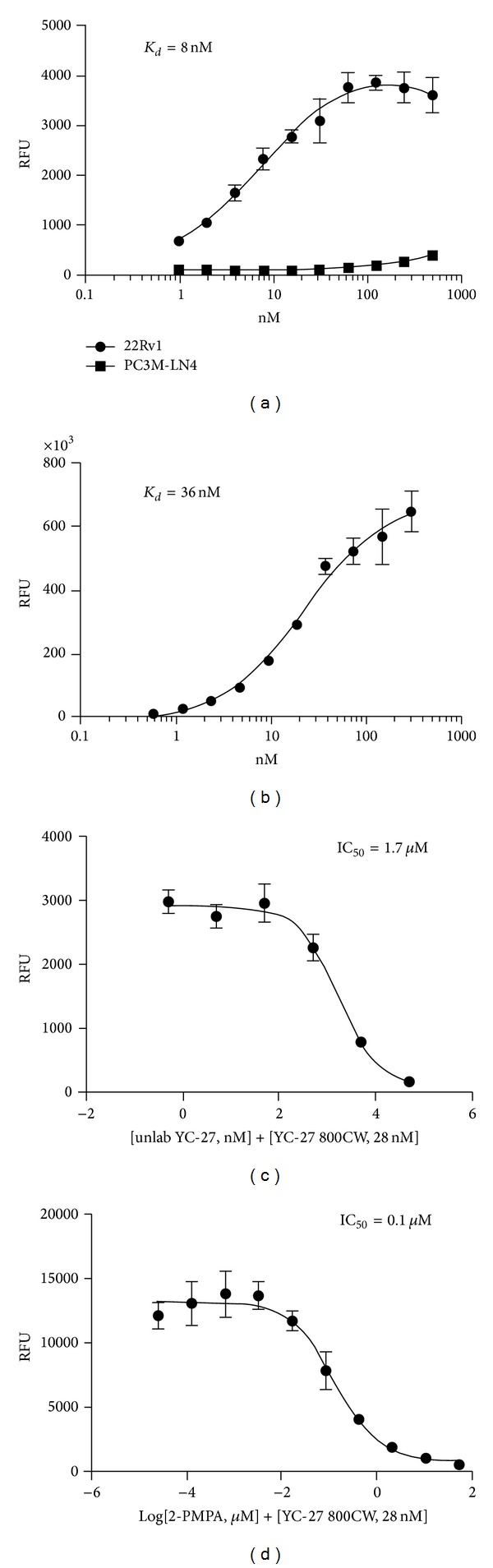
Immunofluorescent cell-based comparison of binding and inhibition. (a) 22Rv1 and PC3 M-LN4 cells were incubated with YC-27 800CW (1 nM to 0.5 *μ*M). Apparent *K*_*d*_ = 8 nM. *K*_*d*_ for PC3 M-LN4 could not be determined. (b) LNCaP cells were incubated with YC-27 800CW (0.5 nM to 0.5 *μ*M, apparent *K*_*d*_ = 36 nM). Competition of YC-27 800CW binding to LNCaP cells by unlabeled YC-27 ((c), IC_50_ = 1.7 *μ*M) or inhibition by 2-PMPA preincubation ((d), IC_50_ = 0.1 *μ*M).

To evaluate the affinity and targeting specificity of YC-27 800CW, we performed cell-based dose response assays. Three human prostate tumor cell lines were chosen based on differing expression of PSMA. YC-27 800CW showed dose dependent increases in fluorescence, indicating high affinity binding to 22Rv1 cells (apparent *K*_*d*_ = 8 nM, Figure 2(a)). No significant signal increase was obtained upon addition of YC-27 800CW to the PSMA-negative cell line, PC3 M-LN4 (Figure 2(a)). Importantly, LNCaP cells, which express levels of PSMA that are higher than those of other commercially available lines, exhibited comparable affinity for YC-27 800CW (apparent *K*_*d*_ = 36 nM, Figure 2(b)) with a significantly higher (~200-fold) fluorescence intensity at saturation, reflecting a higher number of binding sites for the probe on these cells.

Specificity was further confirmed by competition studies. LNCaP cells were treated with YC-27 800CW in combination with either unlabeled YC-27 (Figure 2(c)) or with 2-PMPA, a potent competitive inhibitor of PSMA enzymatic activity (Figure 2(d)). Both compounds inhibited effectively as seen by dose response decreases in signal intensity. The IC_50_ for unlabeled YC-27 and 2-PMPA were 1.7 *μ*M and 0.1 *μ*M, respectively, confirming PSMA as the target for the small molecule.

### 3.3. *In Vivo* Probe Performance

**Figure 3:**
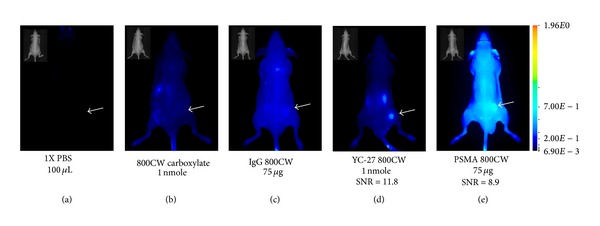
Tumor imaging with YC-27 800CW. SCID Hairless mice implanted with 22Rv1 (right flank) and PC3 M-LN4 (left flank) cells received (a) 1X PBS, 100 *μ*L; (b) 800CW carboxylate, 1 nmol; (c) IgG 800CW, 75 *μ*g; (d) YC-27 800CW, 1 nmol (D/P = 1; SNR = 11.8); or (e) PSMA 800CW, 75 *μ*g (D/P = 1; SNR = 8.9), which was allowed to clear for 24 h prior to imaging on Pearl Impulse Imaging System. Signal is presented in pseudocolor. All images were normalized to the same LUT. White arrows indicate the location of the 22Rv1 tumor. Inset panels show the white light image of the mouse.

Male mice were injected subcutaneously with 22Rv1 cells in the right flank and PC3 M-LN4 cells in the left for direct comparison of PSMA targeting probes. As expected, animals receiving any of the three control compounds 1x PBS, 800CW carboxylate, or IgG 800CW showed minimal retention in either tumor (white arrows, Figures 3(a)–3(c)). Both YC-27 800CW and PSMA 800CW bound the 22Rv1 tumor (white arrows, Figures 3(d) and 3(e), resp.), while little or no signal was visible in the PC3 M-LN4 tumors. As anticipated, animals injected with PSMA 800CW showed incomplete clearance of the probe at the 24 h time point (Figure 3(e)).

**Figure 4:**
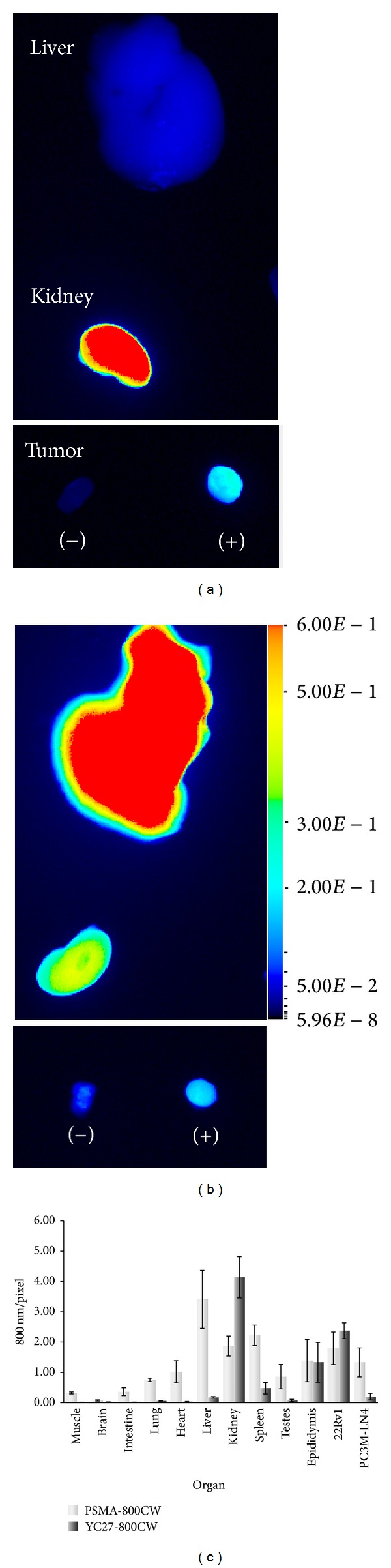
Tissue distribution of fluorescence signal. Tissues were excised at endpoint from animals given YC-27 800CW (column (a)) or PSMA 800CW (column (b)). Representative whole organs (liver, kidney, and tumors) were imaged on the Pearl Impulse and reviewed for residual fluorescence of each probe 24 h after intravenous administration. (c) Tissue sections (8 *μ*m; frozen) were prepared from muscle, brain, intestine, lung, heart, liver, kidney, spleen, testes, epididymis, and tumors, 22Rv1 and PC3 M-LN4. Sections were imaged on Odyssey CLx (21 *μ*m) and 800 nm signal intensity per pixel was plotted for both probes.

Examination of excised liver, kidney, and tumors from animals given YC-27 800CW and PSMA 800CW is presented in Figures 4(a) and 4(b), respectively. As expected, YC-27 800CW showed negligible signal in the liver and high signal in the kidneys, indicating no significant liver retention. PSMA 800CW on the other hand showed the opposite with high fluorescent signal in the liver and low signal in the kidneys indicating a higher liver retention. Both probes were specific for the PSMA-expressing tumor, 22Rv1, with negligible signal in the PC3 M-LN4 tumor. Signals quantified in sections from multiple organs further supported these results (Figure 4(c)). In general, all tissues retained higher levels of PSMA 800CW, including the PC3 M-LN4 tumor, which is consistent with incomplete clearance of the free probe. Both probes accumulated in the epididymis. In considering the higher signal of PSMA 800CW in 22Rv1 tumors, liver, and epididymis, it is important to recognize that the optimal clearance and imaging time point for an antibody-based probe is closer to 72 h rather than the 24 h time point that was optimal for YC-27 800CW [2].

**Figure 5:**
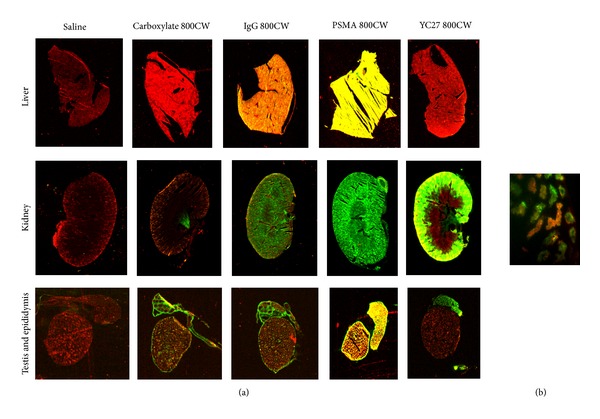
Fluorescence signal retention in specific clearance organs. (a) Tissue sections from mice receiving 1X PBS, carboxylate 800CW, IgG 800CW, PSMA 800CW, and YC-27 800CW were scanned on Odyssey CLx (21 micron). In all images, green indicates signals captured at 800 nm and red represents autofluorescence at 700 nm. Colocalization of the signals is yellow. (b) Higher magnification image (40x) of kidney renal cortex from YC-27 800CW treated mouse.

Sections of liver, kidney, testes, and epididymis, which had higher signal retention, were examined in greater detail (Figure 5). Fluorescence intensity of both control IgG 800CW and specific PSMA 800CW was high in liver sections (Figure 5(a)), consistent with the reported clearance of antibody-based reagents [2]. Low signal was noted in the kidney. YC-27 800CW exhibited significant signal in the kidney, with little or no signal retention in the liver. As noted previously, the epididymis showed residual signal for both PSMA-specific probes and, to a lesser extent, the labeled controls. Interestingly, YC-27 800CW showed strong localized signal in the kidney renal cortex, which houses the proximal tubules. A representative microscopic image of the renal cortex region (40X; Figure 5(b)) showed specific signal in the brush border housing the proximal tubules.

### 3.4. Minimal Effective Dose Determination for YC-27 800CW

**Figure 6:**
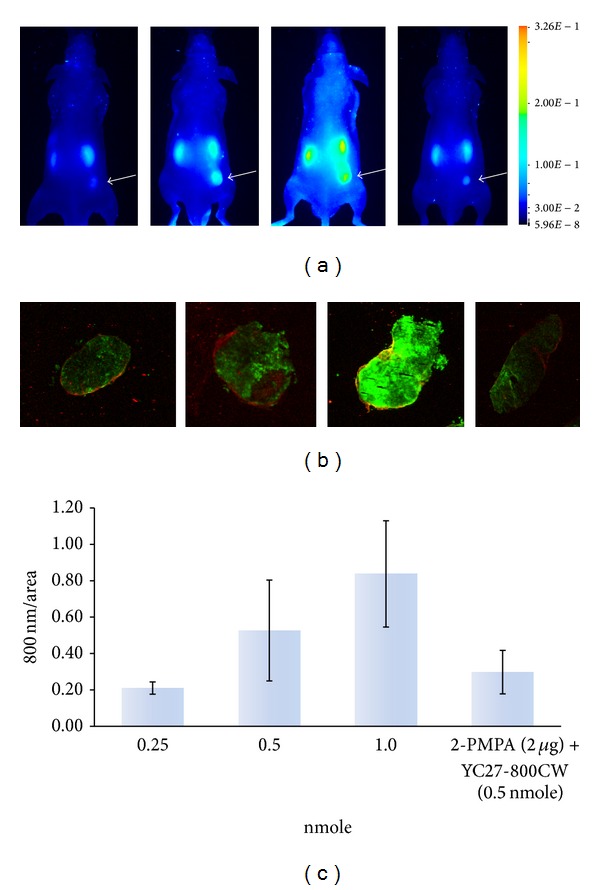
Specificity of YC-27 800CW for tumor imaging* in vivo*. SCID Hairless mice implanted with 22Rv1 (right flank, white arrows) and PC3 M-LN4 (left flank) cells received intravenous injections of 0.25, 0.5, or 1.0 nmol YC27-800CW or 2-PMPA (2 *μ*g) plus YC-27 800CW (0.5 nmol). (a) Animals were imaged intact after 24 hours, using Pearl Impulse; (b) 22Rv1 tumor sections from each animal at endpoint were imaged on Odyssey CLx; (c) signal intensity of each tumor was quantified and normalized to unit area. Intact animal images are presented in pseudocolor. All images are normalized to the same LUT. Odyssey CLx images for tumor sections are shown in green (800 nm) and red (700 nm) and standardized to the same LUT.

SCID Hairless mice bearing 22Rv1 (right flank, white arrows) and PC3 M-LN4 (left flank) xenografts received intravenous injections of 0.25, 0.5, or 1.0 nmol YC-27 800CW or YC-27 800CW (0.5 nmol) plus 2-PMPA (2 *μ*g). Animals were imaged 24 h after injection and tumors excised for further analysis. As shown in Figures 6(a) and 6(b), reasonable tumor-to- background was achieved at the lowest dose of 0.25 nmol. To demonstrate that the observed signals were due to specific binding of the YC-27 800CW probe to PSMA, animals receiving 0.5 nmol of the probe were challenged with 2-PMPA prior to probe injection. The resulting ~50% decrease in signal confirmed the probe bound to its intended target* in vivo*.

## 4. Discussion

YC-27 is chemically optimized from a lead compound that exploited the inhibitory properties of a PSMA-binding urea scaffold [27, 28], which improved pharmacokinetics for detection of prostate and nonprostate tumors [29]. In the current report, we characterized a YC-27 based NIR conjugate and compared its properties to an antibody-based PSMA targeting agent. Our cell-based and* in vivo* data confirmed that the attachment of IRDye 800CW to YC-27 did not alter its target binding characteristics and showed the conjugate bound PSMA-positive cells and tumors with high affinity and specificity. YC-27 800CW produced a strong, specific fluorescent signal in PSMA-positive tumors of intact animals within 24 h, leaving minimal nonspecific background signal.

At the cellular level, PSMA is known to be internalized from its residence at the cell surface via clathrin-coated pits, which are subsequently recycled to the surface to reexpose PSMA [23, 34]. Our cell-based analysis confirmed that YC-27 800CW successfully bound extracellular PSMA, and fluorescence microscopy revealed that significant quantities were specifically internalized by the PSMA-positive cells. The endocytic internalization and recycling of the PSMA target is a mechanism that has afforded significant tumor-targeting sensitivity by other receptor-targeted NIR fluorescent probes, such as IRDye 800CW EGF [35], because the fluorophore label accumulates within the cell while the receptor is returned to the surface for additional probe binding.

YC-27 800CW cleared quickly in intact animals, yielding high tumor-to-background signal within 24 h. In contrast, antibodies are known to have prolonged circulating half-lives [11, 20, 36, 37], so it was not surprising that the antibody-based agent PSMA 800CW did not achieve optimal clearance in this period. The clearance profiles were reflected in the quantitative analysis of fluorescence accumulation in excised organs and tissues, which demonstrated greater signal in all tissues of animals receiving PSMA 800CW. YC-27 800CW fluorescence was most prominent in the renal cortex, particularly in the brush border housing the proximal tubules. Several reports have found PSMA expressed in mouse kidneys [38, 39], but the implications for clinical use are unknown. Basal PSMA expression is also reported in salivary gland, brain, and small intestine [40]. No significant signal was detected 24 h after injection in the prostatic region including the bladder. These distribution patterns support future testing of YC-27 800CW for sensitive and specific distinction between normal, benign, and malignant tissue.

The binding of YC-27 800CW noted in the epididymis may be attributable to recognition of the probe by glutamate receptors (GluR). Previously, it was suggested that GluR may be involved in spermatogenesis, spermatozoa motility, and testicular development [41, 42]. In addition, glutamate binding could be partially displaced by* N*-methyl-D-aspartate in the seminal vesicles [42]. The small molecule, YC-27, has a glutamate component that may be exposed and available for receptor binding. Another potential explanation is that epididymal signals for all labeled agents, probes, and controls may result from a bottleneck in clearance through this highly vascular region coinciding with the imaging time point. Nonetheless, binding to the epididymis is not expected to be problematic in prostate surgery since this tissue is usually removed during prostatectomy.

It has been suggested that the long circulating time exhibited by antibody-based fluorescent contrast agents could be advantageous, because lower doses of the agent would be needed [2, 11, 20, 43]. However, the dose dependence of the small molecule, YC-27 800CW, showed that 22Rv1 tumors were detectable with good tumor-to-background using 0.25 nmol. Thus, rapid clearance of a targeting agent does not compromise its efficacy when the agent has high affinity for the target, and the rapid clearance would be entirely compatible with clinical workflow. This has important implications for translation, since visualization of tumor margins at the primary site, unambiguous identification of tumor-involved secondary sites [12, 13, 20, 44–46], and image-guided real time localization of tumor-involved nodes or residual tissue following resection [14, 47] could potentially benefit long-term patient outcomes.

Currently, ^111^In-capromab pendetide (Prostascint), an antibody that recognizes an intracellular epitope of PSMA, is used as an imaging agent in SPECT. These scans are useful in early phases of diagnosis or postsurgery but may be a disadvantage in the operating suite where exposure to ionizing radiation to patient and surgeon could occur. NIR-labeled optical imaging agents are also gaining attention for tumor resection of a variety of cancers [11, 13, 20, 48, 49]. Due to the cost of clinical trials, single agents with broad reactivity against a number of cancers are desirable because this approach reduces preclinical toxicity work and development of new GMP manufacturing and formulation processes for individual agents [50]. PSMA is broadly expressed on the neovasculature of many solid tumors [3, 4, 21, 51], so agents targeting this protein are good candidates for clinical translation [21, 22], and it may prove valuable to add YC-27 800CW to the toolkit. Use of NIR fluorophore-labeled PSMA targeting agents produced 100% resection with no residual positive margins in PSMA-positive tumor-bearing animals, supporting the use of this approach for prostate tumor resections [15]. Future studies will be needed to define the broad tumor-targeting efficacy of YC-27-based agents for solid tumors.
